# High-risk alcohol consumption is associated with mitochondrial damage-associated molecular patterns in people living with HIV

**DOI:** 10.1016/j.dadr.2025.100359

**Published:** 2025-07-03

**Authors:** Shannon R. Gilstrap, Micheal D. Ho, Joanna M. Hobson, Dyan M. White-Gilliam, Khalid Freij, Michael A. Owens, Shameka L. Cody, S. Justin Thomas, Robert E. Sorge, Burel R. Goodin

**Affiliations:** a Department of Psychology, Samford University, Birmingham, AL, USA; b Heersink School of Medicine, University of Alabama at Birmingham, Birmingham, AL, USA; c Community Dentistry & Behavioral Sciences, University of Florida, Gainesville, FL, USA; d Department of Anesthesiology, Washington University, St. Louis, MO, USA; e Department of Acute, Chronic & Continuing Care, School of Nursing, The University of Alabama at Birmingham, Birmingham, AL 35294, USA; f Department of Psychiatry & Behavioral Neurobiology, University of Alabama at Birmingham, Birmingham, AL, USA; g Capstone College of Nursing, The University of Alabama, Tuscaloosa, AL, USA; h Department of Psychology, University of Alabama at Birmingham, Birmingham, AL, USA

**Keywords:** HIV, Mitochondria, Alcohol use

## Abstract

Alcohol (i.e., ethanol; EtOH) use disorders are common in people with HIV (PWH) and are associated with poor health outcomes. One potential reason for these poor health outcomes in PWH is that alcohol use is associated with mitochondrial damage and dysfunction, potentially leading to cell death. However, the link between alcohol use and mitochondrial functioning in PWH remains unclear. This study specifically investigated the relationship between high-risk alcohol consumption and mitochondrial damage-associated molecular patterns (mDAMPs) in PWH and people without HIV. We analyzed mDAMPs in 122 participants (51 HIV, 71 HIV-) before and after exposure to experimental pain testing to examine mDAMPs reactivity in response to noxious stress. Mitochondrial DAMPs were quantified from serum samples using real-time polymerase chain reaction, providing two variables: ND1 and ND6. High-risk alcohol consumption was assessed using the Alcohol Use Disorders Identification Test – C (AUDIT-C) questionnaire. In this study, we tested whether HIV status influenced the relationship between high-risk alcohol use and mitochondrial damage. Our findings revealed that PWH who engaged in high-risk alcohol consumption exhibited significantly increased mDAMPs following exposure to experimental pain testing for both ND1 (p = 0.045) and ND6 (p = 0.047). These results suggest that the effects of alcohol consumption on mitochondrial damage and dysfunction may be exacerbated by HIV infection. This study highlights the risk of high-risk alcohol consumption on mitochondrial health for PWH.

## Highlights

• High-risk alcohol use elevates DAMPs in people with HIV after pain exposure.
• HIV status moderates the link between alcohol use and mitochondrial damage.
• Experimental pain testing triggered measurable mitochondrial stress markers.

## 1 Introduction

Alcohol use is prevalent among people with HIV (PWH), with studies indicating higher rates of hazardous alcohol consumption compared to the general population (Galvan et al., 2002, Williams et al., 2016). This increased prevalence can be attributed to multiple factors, including psychological distress related to HIV diagnosis, social stigma, and comorbidities such as pain and sleep disturbances. In response, PWH may use alcohol as a coping mechanism (Galvan et al., 2002). Additionally, alcohol use may pose significant unique risks to PWH by potentially undermining adherence to ART, weakening the immune system, and contributing to the progression of HIV disease (Baum et al., 2010, Braithwaite and Bryant, 2010, Williams et al., 2016). Furthermore, alcohol consumption is associated with riskier behaviors, such as unprotected sex, increased likelihood of other substance use/overuse, and reduced healthcare engagement, which can further complicate HIV management and increase the risk of comorbidities (Scott-Sheldon et al., 2013, Shuper et al., 2009).

At the cellular level, alcohol is known to lead to mitochondrial damage by increasing oxidative stress, reducing mitochondrial DNA integrity, and disrupting ATP production (Hoek et al., 2002, Molina et al., 2006). These effects are concerning in the context of HIV infection, where mitochondrial dysfunction is already prevalent due to chronic inflammation, viral replication, and ART-related toxicity (Kallianpur et al., 2020, Swinton et al., 2019). When high-risk alcohol consumption and HIV infection co-occur, they may contribute mitochondrial damage, accelerate cellular injury, and raise the risk of the development of conditions such as liver disease, neurocognitive disorders, and cardiovascular complications (Britton et al., 2021, Kelso et al., 2015, Szabo and Mandrekar, 2009, Zhang et al., 2008).

In recent years, dysfunctional and damaged mitochondrial health has emerged as an area of interest, specifically in attempting to understand the cellular health and regulation of metabolism in PWH. There are a multitude of factors that can lead to dysfunctional and damaged mitochondrial health, such as aging, infections, and even certain types of drugs (i.e., ART) (Cote, 2007; Ganta and Chaubey, 2019; Schank et al., 2021). Much research has shown the manifestations of mitochondrial toxicity in response to adverse ART-associated events (Perez-Matute et al., 2013). For example, research has shown that an increase in the production of tumor necrosis factor-alpha directly induces mitochondrial damage, which has been observed within PWH (Cossarizza et al., 1997). Additionally, it has been demonstrated that ART accelerates mitochondrial aging and induces dysfunction in the mitochondria through mitochondrial DNA mutations (Payne et al., 2011).

HIV itself has also been shown to contribute to mitochondrial damage outside of ART. HIV and polypeptides associated with HIV have been shown to contribute to mitochondrial dysfunction and cause apoptosis in both CD4 +  and CD8 +  T cells (Arnoult et al., 2003, Muthumani et al., 2003, Roumier et al., 2003). A study conducted by Morse and associates provided evidence to support that HIV infection has a direct effect on mitochondria through a decrease in the mitochondrial DNA in adipose tissue and nuclear DNA ratios (Morse et al., 2012). Additionally, they also found that immune cell activation and inflammation were involved in the process (Morse et al., 2012).

However, the interplay between HIV infection and the various factors that influence mitochondrial health, such as alcohol consumption, presents a novel and critical research area. In previous literature, alcohol consumption has been shown to have negative effects on one’s mitochondrial health (Leon et al., 2021; Thoudam et al., 2024). When alcohol is metabolized, it produces toxic byproducts such as acetaldehyde and reactive oxygen species (Bailey and Cunningham, 1998, Cederbaum, 2012, Zakhari, 2006), resulting in oxidative damage and stress and subsequent mitochondrial dysfunction (Guo et al., 2013). As a direct result, the combination of oxidative stress and mitochondrial dysfunction can have implications within the mitochondrial DNA copy number, proper functioning of mitochondrial DNA through genetic mutations, and deletions (Filograna et al., 2021, Ide et al., 2001). While alcohol consumption is a contributor to dysfunctional mitochondrial health and oxidative stress, its effects may be exacerbated or even altered in PWH due to the virus's direct impact on mitochondrial health as well as the side effects of ART (Cote et al., 2002, El-Assal et al., 2004, Molina et al., 2006).

Despite the growing recognition of alcohol's impact on mitochondrial health in the general population, limited research has explored the relationship between alcohol use and mitochondrial functioning in PWH. Understanding how HIV infection influences the effects of alcohol consumption on mitochondrial damage could help us discover the mechanisms driving disease progression and highlight target areas for intervention in PWH. Therefore, we hypothesized that high-risk alcohol use would be significantly and more strongly associated with greater mitochondrial damage (i.e., greater mDAMPs) for PWH relative to people without HIV.

## 2 Methods

The current study was part of a larger parent investigation that examined the impact of insomnia on pain, physical function, and inflammation in PWH (R01HL147603). The parent study collected data, including self-report questionnaires, daily diaries, experimental pain testing, and various biomarkers, during multiple study visits. More details of the findings and procedures from this study can be found elsewhere (Cody et al., 2022, Gilstrap et al., 2023, Gonzalez et al., 2019, Hobson et al., 2022, Owens et al., 2019). However, none of these published studies overlap with the current research. The University of Alabama in Birmingham institutional review board approved all study procedures, and consent was collected from all participants before they participated in the study.

### 2.1 Participants

We recruited both HIV and HIV- participants for this study. The HIV participants were recruited from the UAB 1917 HIV Clinic, which provides comprehensive health care for PWH across Alabama and the greater southeast United States. The HIV- participants were recruited from the greater Birmingham metropolitan area via posted flyers. Interested participants were instructed to call to join the study, and eligibility was determined during a phone screener. The study included participants with and without HIV who were aged 18–85 years. For the PWH, they were required to be on a stable ART regimen. Additionally, an OraQuick Advance Rapid HIV Swab test was performed on the participants who were HIV- to confirm the absence of HIV. All participants were administered the Structured Clinical Interview for Sleep Disorders-Revised by a trained researcher to assess for any possible sleep disorders. Any participants who met the criteria for any additional sleep condition apart from insomnia were excluded from the study. Additional exclusion criteria included medical conditions that could confound experimental variables, such as neurological disorders (i.e., stroke), heart disease, cancer, epilepsy, bipolar disorder, uncontrolled hypertension, circulatory disorders, pregnancy, acute infections, or recent injury/surgical procedure that occurred within the last 6 months. Following enrollment into the study, participants were provided with a unique identification number for use throughout the study. No identifying information was maintained in the dataset. A total of 122 participants were enrolled in the study.

### 2.2 Medical record review

A Medical Record Review was completed to confirm prescribed medications and diagnoses. Medical records were also used to obtain the HIV participants' most recent CD4 T-cell count, their immune status, and the value of their recent viral load that was collected during the study.

### 2.3 Urine drug screen

Participants underwent a CLIA-waived urine drug test to screen for the presence of substance use. The test was conducted using a commercially available, FDA-approved panel that could detect multiple drug classes, including amphetamines, benzodiazepines, cannabinoids, cocaine, opioids, and barbiturates. Testing was conducted according to the manufacturer’s instructions. Results were collected on-site within 5 min by trained researchers. Both positive and negative results were recorded and used to assess recent substance use within the sample. The CLIA-waived urine drug test was indicated to meet the criteria for simple, low-risk testing while ensuring reliable results (Pandya et al., 2023).

### 2.4 Questionnaires

#### 2.4.1 Demographics questionnaire

A demographics questionnaire was completed to collect general participant background information. It was utilized to obtain the participants’ self-reported race, gender, ethnicity, and age.

#### 2.4.2 Alcohol use disorders identification test – C (AUDIT-C)

AUDIT-C is an alcohol screener that is utilized to help identify patients who are high-risk alcohol consumer or may potentially have active alcohol use disorders. It is a modified version of the 10-item AUDIT questionnaire that the World Health Organization developed. The questionnaire contains 3 questions and is scored on a 0–12 scale, where 0 reflects no alcohol use. A score of 4 or more in men is considered positive for high-risk alcohol consumption, whereas a score of 3 or more in women is considered positive for high-risk alcohol consumption. Overall, the higher the AUDIT-C score then the more likely that the patient's drinking may be affecting his/her health and safety.

#### 2.4.3 Penn state cigarette dependence index (PSCDI)

The PSCDI is a brief, 10 question self-report measure that was developed to assess the severity of nicotine dependence among cigarette use. It evaluated multiple dimensions of dependence including cravings and withdrawal. The PSCDI has been proven to be a reliable tool for both clinical and research settings(Borland et al., 2010; Bover et al., 2008; Fidler et al., 2011).

### 2.5 Experimental pain testing

Experimental pain testing is a non-invasive battery of noxious stimuli commonly used as a method to induce and assess physiological reactivity. Experimental pain testing incorporates different types of noxious stimuli, such as temperature, pressure, and touch, that are meant to be painful. Experimental pain testing is a standardized test that can be utilized for both clinical and research purposes (Mucke et al., 2021). These tests involved controlled delivery of painful stimuli to measure responses such as pain threshold and pain tolerance to examine the body’s stress response system. Physical stressors have been found to cause a physiological response that can lead to an increase in oxidative stress which in turn contributes to mitochondrial damage. A stressor is classified as any stimulus that evokes a physiological stress response (i.e., pain or non-pain related) (Hannibal and Bishop, 2014). Previously, we have utilized experimental pain testing successfully as a means of probing mDAMPs reactivity in PWH (Gilstrap et al., 2023). For this study, we utilized experimental pain testing to examine changes in mDAMPs following exposure to the tests.

### 2.6 Mitochondrial DNA damage associated molecular pattern (mDAMP) Assay

Butts and associates previously described the following methodology (Butts et al., 2022). mDAMPs were assessed in serum from human subjects. Blood samples were collected pre- and post-exposure to experimental pain testing. Briefly, cell-free mitochondrial DNA was extracted from 100 µl of serum using a MagMax™ Cell-Free DNA Isolation Kit (Applied Biosystems, USA) following the manufacturer’s instructions with minor adaptations (2.5 µL of the MagMax™ Cell-Free DNA Magnetic Beads per sample was used instead of 5 µL per sample). Cell-Free DNA was eluted in 20 µl volumes and aliquots were stored at −80 °C. mDAMPs were assessed via amplification of sequences within the NADH dehydrogenase subunit 1 (ND1) and NADH dehydrogenase subunit 6 (ND6) regions of the mitochondrial DNA by real-time polymerase chain reaction as previously described with minor modifications using a StepOne Plus Real-Time Polymerase Chain Reaction system (Thermo Fisher Scientific, USA) (Simmons et al., 2013, Yuzefovych et al., 2019). The mDAMPs assay provided two variables to represent markers of damage: ND1 and ND6. Blood was drawn before and after experimental pain testing to examine changes in mDAMPs levels.

### 2.7 Statistical analysis

Descriptive data were calculated and reported as means and standard deviations or numbers and percentages as appropriate. Chi-square was used to examine relationships between categorical variables. An independent *t*-test was utilized to examine relationships between continuous variables. Additionally, a moderation analysis was conducted to examine the strength and direction of the relationship between two variables and how that changed depending on the level of a third variable (the moderator). In this study, we tested whether HIV status influenced the relationship between high-risk alcohol use and mitochondrial damage using PROCESS (v3.4) (Hayes, 2022). Any participants who did not have both samples of mDAMPs were excluded from the moderation analysis. Additionally, due to the parent study examining the impact of insomnia on pain, physical function, and inflammation in PWH, we decided to control for pain status (i.e., chronic pain vs. no pain) and insomnia status (i.e., displayed insomnia symptoms vs did not display insomnia symptoms based on a brief sleep interview). Finally, given that a subset of participants tested positive for cannabis use on the Drug Screener Test and nicotine use through the PSCDI, we controlled for both cannabis and nicotine use to isolate the effects of alcohol consumption on the observed relationship. Cannabis use was selected as a covariate from among the substances assessed in the urine drug screen due to its higher prevalence within our sample. Data was analyzed using SPSS Statistical Software (v28; International Business Machine Corporation, IBM).

## 3 Results

The sample included approximately 54.1 % cisgender women and 42.6 % cisgender men participants (mean age = 44.76 years, SD = 12.72). Approximately 95.9 % of participants identified as non-Hispanic or Latino and the majority of the participants were Black (65.6 %). Table 1, Table 2 shows the demographics and differences in mDAMPs between HIV and HIV groups. Participant age and ethnicity were found to not be significantly different between HIV and HIV- groups. However, gender (p = .01) and race (p < .001) were found to be statistically different between groups.

**Table 1:** Demographics of sample with and without HIV.

	FULL SAMPLE (N = 122)	HIV (n = 51)	HIV- (n = 71)	p
AGE	44.76 ± 12.72	51.71 ± 10.10	39.96 ± 12.16	.07
ETHNICITY				.48
Hispanic/Latino Nonhispanic/Latino	4 (3.3 %) 117 (95.9 %)	1 (2.0 %) 50 (98.0 %)	3 (4.2 %) 67 (94.4 %)	
RACE				<. .001
BLACK/African American WHITE/European Asian/Asian American MULTIRACIAL	80 (65.6 %) 35 (28.7 %) 2 (1.6 %) 5 (4.1 %)	44 (86.3 %) 6 (11.8 %) 0 (0.0 %) 1 (2.0 %)	36 (50.7 %) 29 (40.8 %) 2 (2.8 %) 4 (5.6 %)	
Gender				.01
Cisgender Man Cisgender Woman Transgender, Man to Woman Transgender, Woman to Man	52 (42.6 %) 66 (54.1 %) 3 (2.5 %) 1 (0.8 %)	28 (54.9 %) 20 (39.2 %) 3 (5.9 %) 0 (0.0 %)	24 (33.8 %) 46 (64.8 %) 0 (0.0 %) 1 (1.4 %)	

Note: Numbers in the table are mean with standard deviations or percentages. For comparisons between HIV and HIV- groups, the chi-square test was used for categorical variables and *t*-test for continuous.

**Table 2:** Differences in DAMPs Ratios and AUDIT Scores Between Groups.

	**HIV**	**HIV-**	**P**
**ΔND1**	2.13 ± 3.47	1.74 ± 1.43	.13
**ΔND6**	2.01 ± 5.02	1.26 ±.96	.07
**AUDIT-C**	3.17 ± 2.71	2.45 ± 2.19	.05

Note: Numbers in the table are mean with standard deviations. For comparisons between HIV and HIV- groups, an independent *t*-test was conducted. ND1 =  NADH dehydrogenase subunit 1 ND6 =  NADH dehydrogenase subunit 6

This study found that HIV participants scored significantly higher on the AUDIT questionnaire in comparison to the HIV- participants (*t*(110) = -1.55, p = .05), meaning HIV participants consumed higher amounts of alcohol on average. However, there was no statistically significant difference between DAMP changes across exposure to experimental pain testing. Additionally, Table 3 presents the number of participants who tested positive for substances on the urine drug screen. The most detected substances were cannabis (n = 35) and cocaine (n = 16). Lastly, out of 122 participants, 40 stated that they smoked cigarettes and completed the PSCDI. Out of the 40 participants who completed the PSCDI***.***

**Table 3:** Frequencies of Positive Urine Drug Screens by Substance.

Substance	Full Sample (n = 114)
Cocaine	**16 (13.1 %)**
Cannabis	**35 (28.7 %)**
Ecstasy	**0 (0 %)**
Methamphetamine	**2 (1.6 %)**
Methadone	**2 (1.6 %)**
Morphine	**2 (1.6 %)**
Oxycodone	**2 (1.6 %)**
Phencyclidine	**0 (0 %)**
Amphetamine	**4 (3.3 %)**
Barbiturates	**2 (1.6 %)**
Benzodiazepines	**1 (0.8 %)**
Buprenorphine	**4 (3.3 %)**

Note: We had missing data for 8 participants, bringing our sample for the urine drug screen from 122 to 114.

Note: ND1 =  NADH dehydrogenase subunit 1

**Fig. 1:**
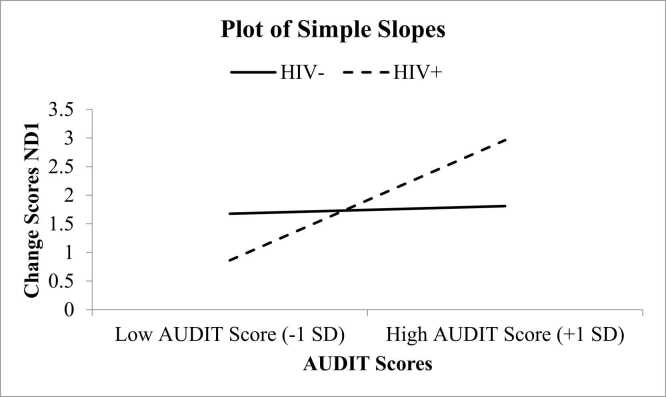
Examination of Moderation Effects of HIV Status on AUDIT Scores and Changes in ND1. Notes: ND6 =  NADH dehydrogenase subunit 6.

**Fig. 2:**
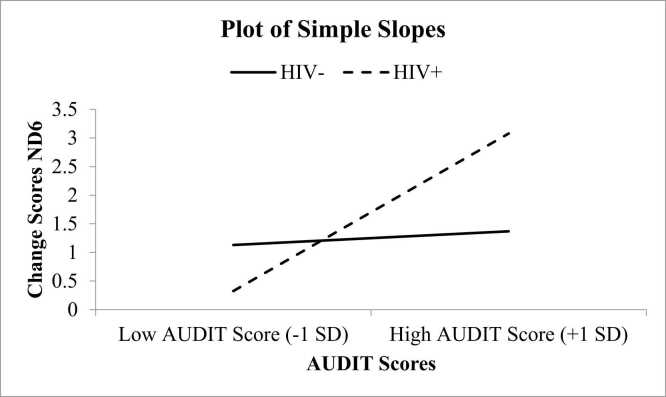
Examination of Moderation Effects of HIV Status on AUDIT Scores and Changes in ND6. Notes: ND6 =  NADH dehydrogenase subunit 6.

These analyses explored how HIV status affects the relationship between AUDIT scores and mDAMPs levels (i.e., ND1 and ND6). Covariates, including pain status, insomnia status, and cannabis use, were also accounted for in these analyses. In the first model (Fig. 1), examining ND1 (a type of mDAMP), HIV status accounted for approximately 16 % of the variation in alcohol use scores (R² =.16, F(7, 97) = 2.70, p = .013). Additionally, the way HIV status interacted with alcohol use added nearly 4 % more explanation (ΔR² =.04, F(1, 97) = 4.14, p = .045), meaning that HIV status slightly influenced how alcohol use affected ND1 levels in response to stress (B =.41, SE =.20, t = 2.03, p = .045). A similar pattern emerged in the second analysis (Fig. 2), which focused on ND6. HIV status explained about 16 % of the variation in alcohol use scores (R² =.16, F(7, 97) = 2.59, p = .017), with its interaction with alcohol use contributing another 3.8 % to the explanation (ΔR² =.035, F(1, 97) = 4.05, p = .047). This confirmed that HIV status also played a role in how alcohol use affected ND6 levels after stress (B =.54, SE =.27, t = 2.01, p = .047). These findings suggest that HIV status exacerbates the impact of alcohol consumption on mDAMPs following exposure to experimental pain testing. Specifically, PWH who score higher on the AUDIT questionnaire have an increase in mDAMPs following exposure to experimental pain testing.

## 4 Discussion

The results of this study provided support for the relationship between high-risk alcohol consumption, mitochondrial damage, and HIV status. The findings of this study demonstrated that HIV status moderated the relationship between high-risk alcohol consumption and mitochondrial damage, evident by the changes that occurred in mDAMPs. Specifically, the study showed that PWH who engaged in high-risk alcohol consumption exhibited increased mDAMPs following exposure to experimental pain testing (i.e., changes in ND1 and ND6).

This study builds upon existing research that examines the effects of alcohol and HIV on mitochondrial health, specifically damage and dysfunction. Previous studies have investigated the independent impact of alcohol consumption and HIV status on mitochondrial damage (Hoek et al., 2002; Hulgan and Gerschenson, 2012). For example, prior literature has highlighted the role of alcohol-induced oxidative stress and its effect on the integrity of mitochondrial DNA and ART-related mitochondrial toxicity in PWH (Guo and Ren, 2010, Mallon et al., 2005). Additionally, our findings were similar to research conducted by Morse et al., which examined mitochondrial damage in untreated HIV patients as well as ART-induced changes in mitochondrial health (Morse et al., 2012). However, this study provides novel insight into the examination of HIV status and high-risk alcohol consumption combined impact on mitochondrial health, indicated by mDAMPs levels. This study contributes to the understanding of how high-risk alcohol consumption can exacerbate mitochondrial damage in PWH in comparison to HIV-negative individuals. By examining the combined impact of HIV status and high-risk alcohol use, this study offers a novel perspective on the multifaceted nature of mitochondrial health.

Some mechanisms potentially underlie the observed moderation effects shown in this study. Chronic alcohol use has been shown to produce reactive oxygen species and acetaldehyde, which in turn can lead to oxidative stress and mitochondrial damage (Guo and Ren, 2010). Additionally, HIV infection has been found to contribute independently to mitochondrial damage and dysfunction through chronic inflammation, the activation of the immune system, and ART-related toxicity (Mallon et al., 2005, Morse et al., 2012). As shown in this study, the combined effects of high-risk alcohol consumption and HIV may amplify mitochondrial damage and dysfunction. Lastly, mitochondrial damage has been associated with increased levels of pro-inflammatory cytokines, such as tumor necrosis factor-α, which could potentially further exacerbate cellular injury in PWH (Pinti et al., 2010).

Several limitations are present in this study. First, due to the study being cross-sectional, it in turn limits the ability to infer causality. If we were to repeat the study in the future, conducting a longitudinal study would allow us the ability to better understand the relationship between high-risk alcohol consumption, mitochondrial damage, and HIV status. Additionally, the study relied on self-reported alcohol consumption by the participants, which can be subject to several biases, such as recall and social desirability bias. Also, the study consisted of a smaller sample size, and our findings may not generalize to all populations of PWH. Lastly, although data on cigarette smoking and urine drug screening were included, the potential influence of unmeasured substance use not accounted for in the analysis may have affected mitochondrial function or interacted with alcohol use and HIV status, thereby potentially influencing the observed outcomes. In the future, research should explore the longitudinal impact of high-risk alcohol consumption on mitochondrial damage in PWH across a larger sample of the population. Additionally, studies examining the interactions between other substance misuse and mitochondrial health could further enhance our understanding of the challenges that are faced by PWH.

In conclusion, this study provides evidence of the harmful effects of high-risk alcohol consumption on mitochondrial health in PWH following exposure to experimental pain testing. By demonstrating that HIV status could potentially exacerbate alcohol-induced mitochondrial damage, the results of this study emphasize the importance of addressing alcohol use as a key component of HIV care. Identifying these relationships may help clinicians develop tailored treatment strategies that address alcohol use and mitochondrial health, ultimately improving the quality of life and health outcomes for PWH.

## CRediT authorship contribution statement

**Micheal D. Ho:** Writing – review & editing, Investigation. **Shannon R. Gilstrap:** Writing – review & editing, Writing – original draft, Investigation, Formal analysis, Conceptualization. **Dyan M. White-Gilliam:** Writing – review & editing, Supervision, Project administration, Investigation. **Joanna M. Hobson:** Writing – review & editing, Investigation. **Khalid Freij:** Writing – review & editing. **Shameka L. Cody:** Writing – review & editing, Investigation. **Michael A. Owens:** Writing – review & editing. **Robert E. Sorge:** Writing – review & editing, Supervision, Funding acquisition. **S. Justin Thomas:** Writing – review & editing. **Burel R. Goodin:** Writing – review & editing, Supervision, Funding acquisition, Formal analysis, Conceptualization.

## Declaration of Competing Interest

The authors declare the following financial interests/personal relationships which may be considered as potential competing interests: Burel Goodin reports financial support was provided by National Heart Lung and Blood Institute. If there are other authors, they declare that they have no known competing financial interests or personal relationships that could have appeared to influence the work reported in this paper.
